# Group B Streptococcal Subscapular Abscess: A Case Report and Review of Literature

**DOI:** 10.7759/cureus.82363

**Published:** 2025-04-16

**Authors:** Yassir P Hussain, Roy Abraham, Muhammad Thahir, Roshan Pais, Ahmed Elnaggar, Abdul M Sathar, Khalid Al Hamadi

**Affiliations:** 1 Department of Orthopedic Surgery, Dubai Hospital, Mohammed Bin Rashid University of Medicine and Health Sciences (MBRU), Dubai, ARE

**Keywords:** abscess drainage, abscess in elderly, chest wall abscess, group b streptococcus (gbs), hematoma post physiotherapy, infected hematoma, invasive group b streptococcus infection, streptococcus agalactiae, subscapular abscess, subscapular muscle sparing approach

## Abstract

Subscapular abscesses are rare, and delayed diagnosis is common. Diagnosis is difficult as it often presents with mild symptoms like stiffness and muscular pain, mimicking other more common pathologies. Delayed diagnosis can lead to the spread of infection and increased morbidity and mortality. A high index of suspicion, prompt imaging, and early surgical drainage are keystones of management. Though all age groups are known to be affected, our case is the oldest reported patient at 83 years. He developed an abscess following a hematoma due to manipulation during physiotherapy, which subsequently got infected. Ultrasonogram and MRI demonstrated the abscess in the subscapular space. Culture and microscopy of the aspirate revealed the causative organism to be *Streptococcus agalactiae*, a Group B Streptococcus (GBS), which has not previously been reported as a causative organism. Invasive GBS (IGBS) in the elderly is a growing concern. We opted to drain the collection using a muscle-sparing, single-incision posterolateral approach. A literature review yielded 26 cases, which were then analyzed for age, sex, risk factors, clinical presentation, imaging, bacteriological profile, antibiotic use, and surgical approaches.

## Introduction

Subscapular abscesses, defined as abscesses involving the subscapularis muscle or the space between the subscapularis muscle and the chest wall, are rare and difficult to diagnose [1-8]. Delayed diagnosis can lead to serious complications, including pneumonia (2/27), meningitis (1/27), septicemia (1/27), and even death (1/27) [2-6,8,9]. We describe a case of subscapular abscess in the oldest patient to date with a previously unreported causative organism drained using a muscle-sparing approach, leading to complete recovery without recurrence. A literature review to identify common features and possible management approaches is also presented.

## Case presentation

An 83-year-old male presented to the emergency department with a one-month-old swelling in the right scapular region. It appeared after a physiotherapy session and gradually increased in size. There was no associated pain, fever, or cough. There was no history of any other recent infection, surgeries, injections, intravenous drug abuse, or trauma other than the physiotherapy session. The patient had a history of diabetes mellitus, hypertension, atrial fibrillation, congestive heart failure, cerebral vascular accident, peripheral vascular disease, and dementia and was fully dependent on nursing care. He spoke rarely and was incapable of feeding himself. He was on Apixaban for his atrial fibrillation besides other medications.

**Figure 1:**
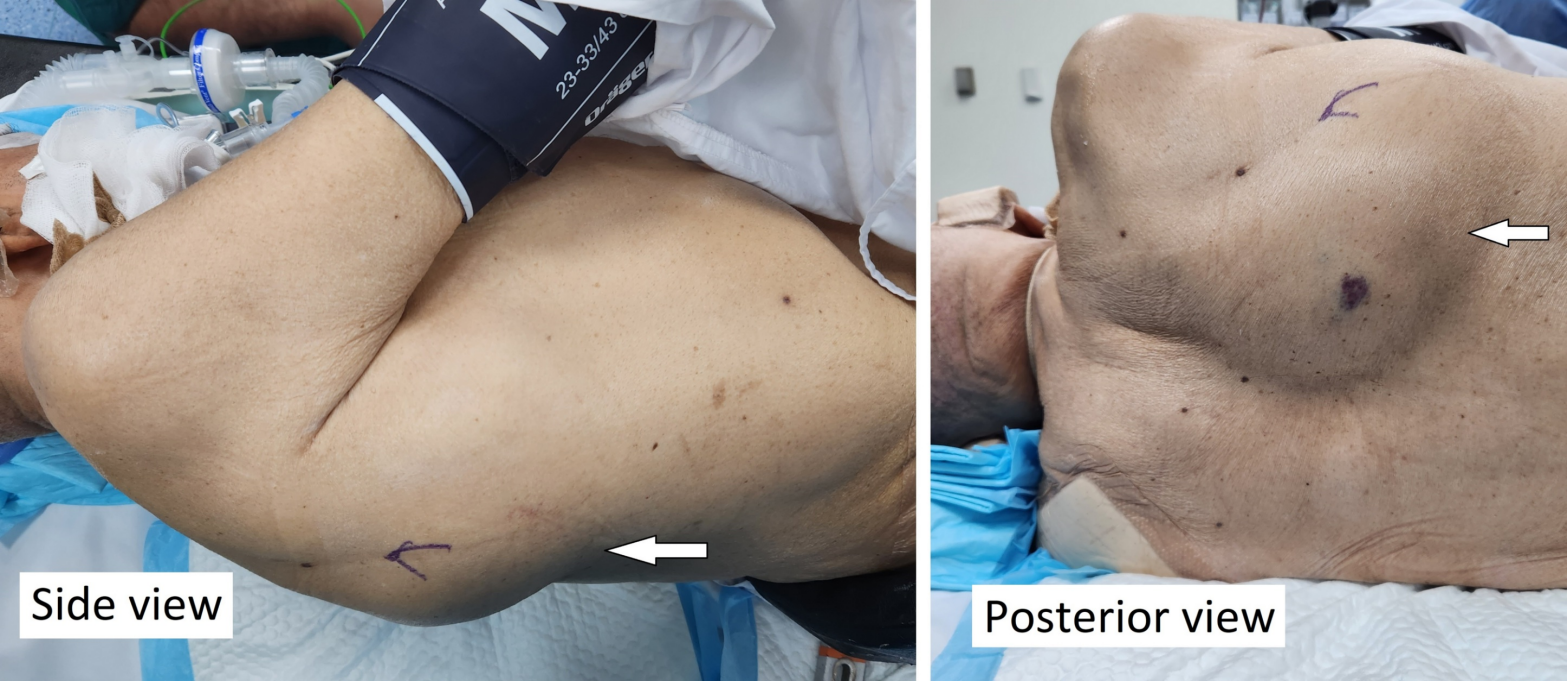
Periscapular swelling on the right side.

On examination, his vitals were normal. There was a large, right-sided, crescent-shaped periscapular swelling, 10 cm in size vertically, 15 cm horizontally, and 5 cm wide, situated around the medial, inferior, and lateral borders of the right scapula (Figure 1). There was no local rise of temperature or tenderness. It was smooth surfaced with well-defined margins, soft in consistency, and fluctuant. There were no skin lesions. Even though the patient was responsive to pain, he did not exhibit any pain on movement of the right upper limb or during palpation.

**Figure 2:**
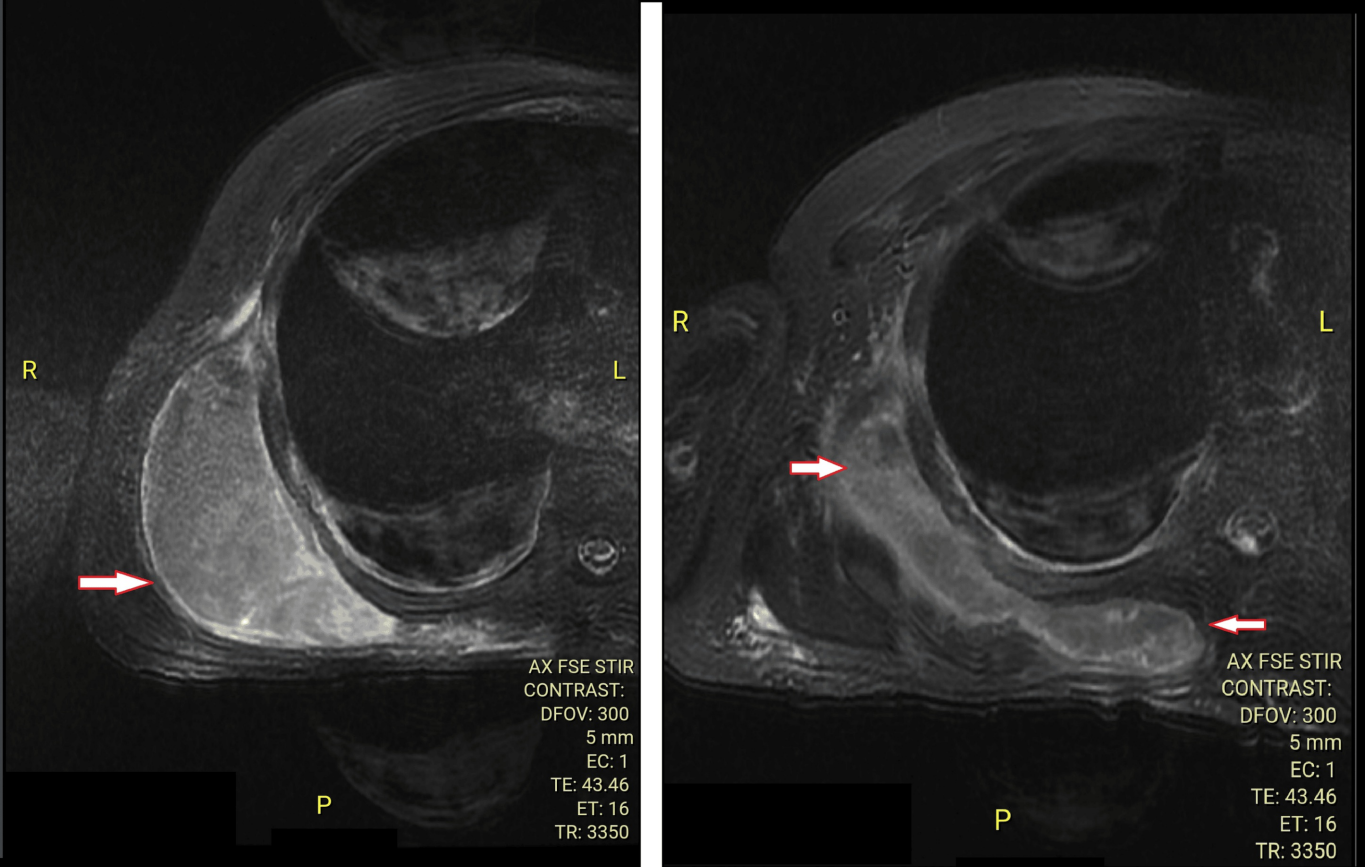
MRI chest with contrast, axial view, showing the subscapular abscess.

On workup, he was found to have elevated blood glucose levels at 141 mg/dL, a total count of 13,000 /µL with 70.8% polymorphs, ESR of 45 mm/first hour, C-reactive protein (CRP) 94.3 mg/L, and procalcitonin of 0.16 ng/mL, suggestive of infection. The enzyme-linked immunosorbent assay (ELISA) for HIV was negative. Ultrasound of the swelling showed a right-sided subscapular collection 9.69 cm x 3.5 cm, reported as a possible hematoma, with no debris within. Aspiration yielded 40 mL of thick, yellowish pus. Cell count could not be performed due to the thickness of the pus, and the smear showed numerous distorted cells with a high concentration of polymorphs. A multiplex polymerase chain reaction (PCR) of the pus showed *Streptococcus agalactiae* (Group B streptococcus). Pus culture for pyogenic bacteria and acid-fast bacilli, as well as PCR for *Mycobacterium tuberculosis*, were negative. The chest X-ray was normal. MRI of the chest and spine showed a right subscapular abscess of size 14 cm x 10 cm x 9 cm, with hemosiderin deposition suggestive of a hematoma (Figure 2). The bulk of the abscess was situated in the subscapular space between the subscapularis muscle and the chest wall, pointing laterally. The subscapularis muscle, glenohumeral joint, rib cage, and spine were normal. The right lung showed mild pleural effusion with consolidation.

**Figure 3:**
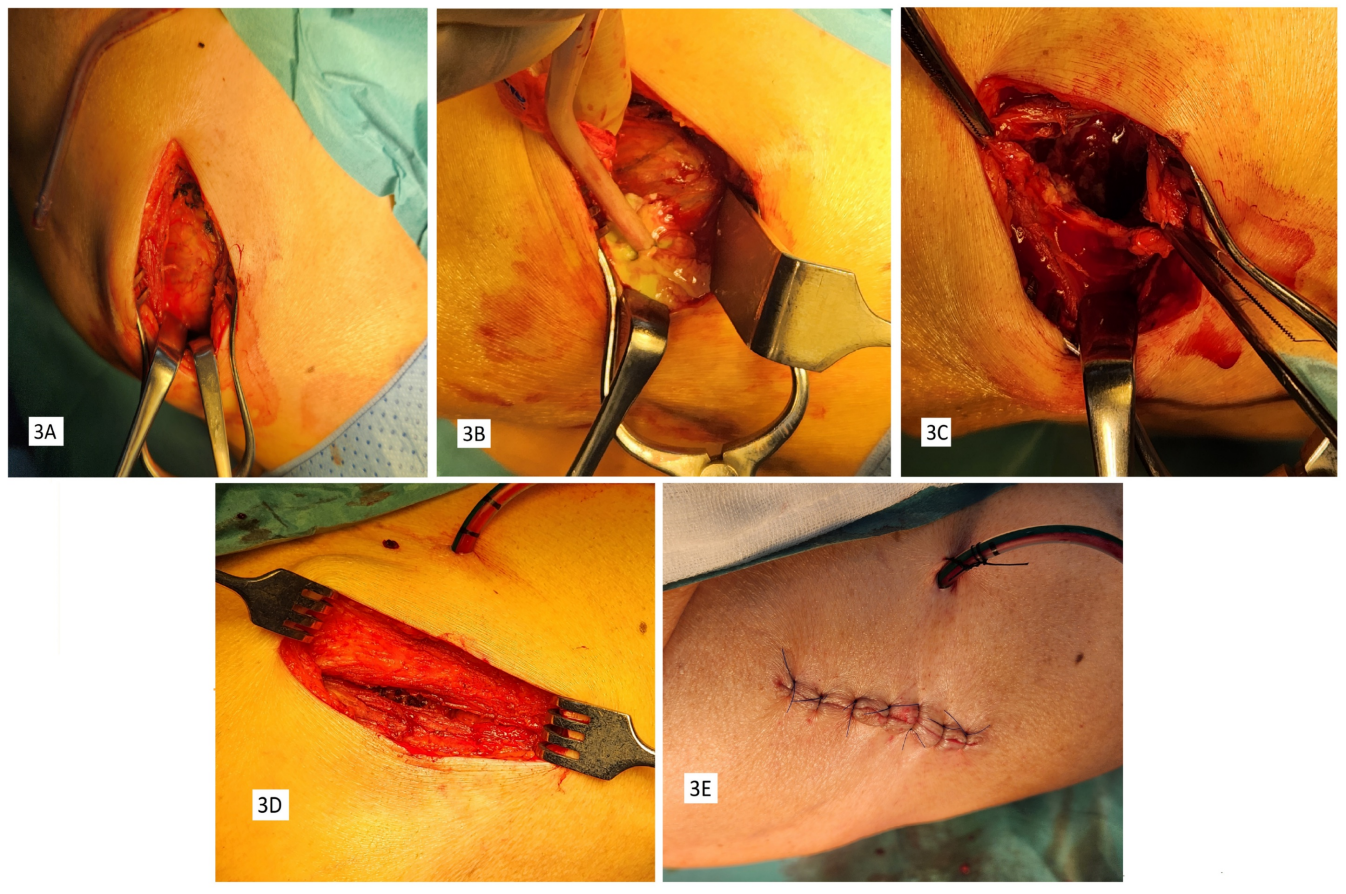
Intra-operative images: (A) abscess sac after dissection; (B) opened abscess cavity with pus; (C) debrided and cleared abscess sac; (D) muscle-sparing approach demonstrated; (E) closure with wide bore drain in situ.

The patient was started on 1 g of intravenous ceftriaxone every 12 hours and prepared for surgery. Based on the abscess location, we decided to use a modification of the posterolateral approach to the subscapular space, as described by Christman-Skieller et al. [10]. The patient was placed in a left lateral position, and through a 15-cm-long incision parallel to the inferolateral border of the scapula, the plane between the teres major and latissimus dorsi muscles was developed, creating a window into the subscapular space. A well-defined abscess wall was identified (Figure 3A), and opening it yielded 200 mL of thick yellow pus (Figure 3B). There were fibrous septa within the abscess cavity. The abscess wall was completely excised using a combination of sharp dissection, curettage, and rongeurs (Figures 3C, 3D). The chest wall and the anterior surface of the subscapularis muscle were debrided. Since the patient was not very muscular, it was possible to access the most medial extent of the cavity. The subscapularis muscle and the chest wall appeared healthy. The pus and tissue samples were sent for culture and sensitivity. All surfaces were thoroughly washed with normal saline, followed by betadine solution, and then again with normal saline. The wound was closed in layers over a large-bore vacuum drain (Figure 3E).

The pus and biopsy cultures were positive for *Streptococcus agalactiae, *which was sensitive to ceftriaxone. The same organism was also isolated from the urine culture and sensitivity. One gram of intravenous ceftriaxone was administered every 12 hours for 17 days. The vacuum drain was removed after 48 hours. His postoperative period was uneventful, and sutures were removed on the 12th day. He was discharged on day 17, and his CRP and procalcitonin were normal, and he was doing well. He continued follow-up in the clinic, and more than a year after surgery, there has been no recurrence of the subscapular abscess.

## Discussion

A PubMed search was conducted using the term “subscapular abscess” for English articles. These were then reviewed by two different authors, and relevant articles were used to build a core collection of 14 articles. These were then fed into Connected Papers, a visual search engine based on the Semantic Scholar database that displays related academic papers in a graph-based interface using co-citation and bibliographic coupling [11]. The resulting papers were again reviewed by two authors, yielding a total of 23 papers representing 26 cases [1-10,12-24]. Data from these papers were then tabulated (Table 1), and the authors' case was added to give a total of 27 patients whose demographics, case characteristics, and treatment methods (Table 2) were analyzed to identify patterns that would help in diagnosis and management.

**Table 1:** Demographic distribution and clinical presentation in the reviewed literature. DM, diabetes mellitus; ESRD, end-stage renal disease; AVF, arteriovenous fistula

Author (Year)	Age	Sex	Risk factors and causes	Duration of symptoms	Fever	Local signs
San Joaquin and Kimball (1980) [23]	1.25	F	None	5 days	Present	Present
Handorf (1983) [5]	19	M	Illicit drug abuse, pneumonia, trauma	6 days	Present	Present
Nowinski and Duchene (2004) [1]	53	M	None	14 days	Present	Present
Saxena et al. (2008) [8]	42	M	DM	Not specified	Present	None
Babayiğit et al. (2009) [12]	7	M	Blunt trauma	21 days	Present	None
Yilmaz and Standard (2012) [9]	9	F	DM	7 days	Present	None
Giugale et al. (2015) [14]	9	F	None	7 days	Present	None
Koratala et al. (2017) [18]	51	M	DM, ESRD with AVF	2 days	Not specified	Not specified
Christman-Skieller et al. (2017) [10]	23	F	IV drug abuse, Hepatitis C	4 days	Present	None
Mourkus et al. (2018) [7]	7	M	None	1 day	None	None
Jagernauth et al. (2018) [17]	38	F	None	4 days	Present	None
Patel et al. (2018) [20]	38	F	Blunt trauma	4 days	Present	None
Khaw and Faisham (2019) [24]	13	F	Blunt trauma	9 days	Present	None
Furuhata et al. (2019) [4]	67	F	Meningitis	2 days	Present	None
Fernández Pérez et al. (2020) [13]	44	M	None	10 days	Present	Present
Park and Jang (2020) [19]	32	F	None	6 months	None	None
East et al. (2020) [2]	47	F	DM	6 days	Present	None
Ham and Wang (2021) [16]	72	M	Atypical pneumonia	2 weeks	Present	None
Ren et al. (2021) [22]	38	M	Diabetes	5 months	Present	Present
Indra et al. (2022) [3]	11	M	Fall from height	7 days	Present	Present
McFarlane et al. (2023) [6]	20	F	Recent submandibular cyst	3 days	Present	None
Author case (2023)	83	M	DM, trauma, urinary infection	1 month	None	None
Pushpasekaran et al. (2024) [21]	49	M	Trauma	14 weeks	Present	Present
Pushpasekaran et al. (2024) [21]	64	M	None	3 days	Present	Present
Pushpasekaran et al. (2024) [21]	53	F	DM, trauma	8 days	Present	Present
Pushpasekaran et al. (2024) [21]	69	M	HIV, COVID-19	7 days	Present	Present
Grange et al. (2024) [15]	45	F	None	1 month	Present	Present

**Table 2:** Investigations and interventions in the reviewed literature. N, neutrophils; L, lymphocytes; M, monocytes; MRI, magnetic resonance imaging; CT, computed tomography; USG, ultrasonogram; MRSA, methicillin-resistant *Staphylococcus aureus*

Author (Year)	WBC (cells/mm^3^)/Differential	CRP (mg/L)	Imaging studies	Glenohumeral joint involvement	Microbiology	Antibiotics used	Duration of antibiotics used	Surgical approach and no. of debridement
San Joaquin and Kimball (1980) [23]	22,500/N62L32	Not specified	Not done	N	Hemophilus influenza type B	Cefazolin, then ampicillin	Not specified	Direct axillary/1
Handorf (1983) [5]	8,700	Not specified	Not done	Y	Staphylococcus aureus	Cephalothin and gentamycin	2 days	None
Nowinski and Duchene (2004) [1]	22,000/N79L2M8	142	MRI	N	Staphylococcus aureus	IV vancomycin (2 days), then IV Nafcillin 2g q4h (8 days) and oral dicloxacillin (11 days)	3 weeks	Posterior inferomedial/1
Saxena et al. (2008) [8]	40,800/N94	Not specified	MRI	N	Staphylococcus aureus	Vancomycin + Meropenem empirical, Flucloxacillin (IV 3 weeks/oral 3 wks) and Rifampicin	6 weeks	Posterior inferomedial/1
Babayiğit et al. (2009) [12]	18,900/ N80L10M10	200	MRI	N	No growth	Ampicillin-Sulbactam oral empirical, Ceftriaxone and Vancomycin (IV) 3 weeks	3 weeks	Not specified
Yilmaz and Standard (2012) [9]	21,700/N82L6M6	>100	CT/MRI	Y	MRSA	Ceftriaxone empirical. Linezolid (3 weeks) and then Clindamycin oral (3 weeks)	6 weeks	Deltopectoral/1
Giugale et al. (2015) [14]	21,200/N84L5M7	167	MRI/CT	N	MRSA	Vancomycin empirical then IV Clindamycin	6 weeks	Modified Judet or posterior inferomedial/2
Koratala et al. (2017) [18]	Not specified	Not specified	CT contrast	N	MRSA	Not specified	Not specified	-
Christman-Skieller et al. (2017) [10]	12,890	166	CT	N	MRSA	Vancomycin + Aztreonam + Clindamycin + Levofloxacin empirical, later IV Vancomycin and Ceftriaxone	6 days	Posterior lateral/2 inferomedial/3
Mourkus et al. (2018) [7]	11,000	77	US/MRI/CT	N	Staphylococcus aureus	Flucloxacilin (IV 3 days and oral 3 weeks)	24 days	Posterior inferomedial/2
Jagernauth et al. (2018) [17]	11,100	221	MRI	N	PVL-positive Staphylococcus aureus	Co-amoxiclav IV, oral flucloxacillin	4 weeks	Deltopectoral/1
Patel et al. (2018) [20]	11,100	233	MRI	N	PVL-positive Staphylococcus aureus	IV Flucloxacillin 1g q6h 2 weeks	15 days	Deltopectoral/1
Khaw and Faisham (2019) [24]	27,000	106	US/CT	N	Staphylococcus aureus	Oral Cloxacillin	3 weeks	Posterolateral approach, single incision/1
Furuhata et al. (2019) [4]	16,700	296	CT/MRI	Y	Streptococcus pneumoniae	IV Ceftriaxone, Vancomycin, Ampicillin 1 week empirical, later Ceftriaxone only 2 weeks	3 weeks	Deltopectoral and Subscapular Tenotomy
Fernández Pérez et al. (2020) [13]	-	236	CT	Y	MRSA	Moxclav 1g q8h empirical, then Cefazolin 1g q8h+ Gentamycin 250 mg q12h, after culture IV Cloxacillin 3 weeks and oral Levofloxacin 15 days	36 days	Deltopectoral/1
Park and Jang (2020) [19]	9,740	10	MRI	N	No growth	Not specified	Not specified	Deltopectoral, Radical excision of subscapularis/1
East et al. (2020) [2]	21,900	462	CT	N	Staphylococcus aureus	IV Gentamycin and Co-amoxiclav for 3 days, then IV Flucloxacillin for 7 days, then Oral Clindamycin for 4 weeks	38 days	Deltopectoral /1
Ham et al. (2021) [16]	4,690	82	CT/MRI	Y	Beta-lactamase Escherichia coli	IV Ceftazidime	4 weeks	Deltopectoral/1
Ren et al. (2021) [22]	-	Not specified	X-ray/CT	N	Blastomycosis	IV Amphotericin 1 week and oral Voriconazole 1 year	1 year, 1 week	Percutaneous USG-guided
Indra et al. (2022) [3]	18,800	1,264	US/CT	N	Staphylococcus aureus	Cloxacillin empirical, later Cloxacillin (IV for 11 days and oral 6 weeks)	53 days	Percutaneous USG-guided
McFarlane et al. (2023) [6]	17,460	442	MRI	Y	MRSA	Flucloxacillin empirical, later Clindamycin, Cefazolin Vancomycin preop. Post-op IV Vancomycin 2 weeks and Oral Clindamycin 6 weeks	8 weeks	Deltopectoral/1
Author case (2023)	13,000	94.5	USG/MRI	N	Group B streptococcus. Streptococcus agalactiae	IV Ceftriaxone 1 g 12 h for 17 days	17 days	Posterolateral tendon sparing/1
Pushpasekaran et al. (2024) [21]	Elevated	Elevated	MRI	N	Staphylococcus aureus	IV Vancomycin 2 weeks, Oral Clindamycin 4 weeks	6 weeks	Dual anterior, neck percutaneous/1
Pushpasekaran et al. (2024) [21]	23,000	78	USG/MRI	N	No growth	IV Vancomycin 2 weeks, Oral Clindamycin 4 weeks	6 weeks	Dual anterior, arm also/1
Pushpasekaran et al. (2024) [21]	Not specified	Not specified	USG/MRI	N	Staphylococcus aureus	IV Vancomycin 2 weeks, Oral Clindamycin 4 weeks	6 weeks	Dual anterior/1
Pushpasekaran et al. (2024) [21]	Not specified	Not specified	MRI	N	No growth	IV Vancomycin 2 weeks	Not specified	Deltopectoral and arm/1
Grange et al. (2024) [15]	32,870	263	USG/MRI	Y	Staphylococcus aureus	4.5 g Piperacillin Tazobactam TID + 1.25 g Vancomycin TID initially, 2 g Cefazolin TID + 450 Clindamycin TID 3 weeks	Not specified	Deltopectoral/4 , arthroscopy/3

Patients of all ages were affected, most falling between 20 and 55 years (14/27 patients, constituting 51.85%). The youngest was 1.25 years old, and the oldest was 72 years. Males (14/27, 51.85%) were more affected than females (13/27, 48.14%). About risk factors, 19/27 (70.38%) had at least one risk factor, 6/27 (22.22%) had multiple risk factors, and 8/27 (29.62%) had no risk factors. The most common risk factors were trauma (25.92%), diabetes mellitus (22.22%), pneumonia (7.4%), and drug abuse (7.4%), followed by HIV, COVID-19, chronic kidney disease, meningitis, and urinary tract infection at 3.7% each.

Of the 27 patients, 14 (51.85%) presented with a symptom duration of less than 8 days, peaking at 4 to 7 days. The remaining had symptom durations between 10 days and 6 months. They presented with fever in 23/27 (85.19%), while 3/27 (11.11%) had none. Local signs were present in only 11/27 (40.74%), while 15/27 (55.56%) of patients had none.

On laboratory workup, total white cell count and CRP were elevated in 19/27 (70.37%) of the patients. The most common imaging modalities were MRI (18/27, 66.66%) and CT (12/27, 44.44%). Ultrasound was used in 7/27 (25.92%) cases, and multiple imaging modalities were used in 11/27 (40.74%) cases. The vast majority of cases (20/27, 74.07%) had no glenohumeral joint involvement. The most common causative organism was Staphylococcus (18/27, 66.67%), with *Staphylococcus aureus* being the most frequent (10/27, 37.04%), followed by methicillin-resistant *Staphylococcus aureus* (MRSA) (6/27, 22.22%). No organism could be isolated in 14.81% of the cases.

Vancomycin and clindamycin were the most common antibiotics used for staphylococci, whereas ceftriaxone was the most used for streptococci. The duration of antibiotic therapy was generally 3 to 6 weeks, while for MRSA, it was 5 to 8 weeks.

Surgical drainage was performed in 23/27 (85.18%) cases, percutaneous drainage in 3/27 (11.11%), while 1 patient died before drainage. A wide variety of approaches have been used, evolving over the years. The various surgical approaches, as seen in Table 2, can be classified into axillary [23], posterior [1,7,8,10,14,24], and anterior approaches [2,4,6,9,13,15-17,19-21].

Multiple debridement was done in 5/27 (18.51%) cases [7,10,14,15,21]. The outcome was good in 25/27 (92.59%), with only two patients succumbing to infection: one due to septicemia within 24 hours of admission [5] and the other due to COVID-19 pneumonia and complications [21].

Diagnostic delays are common with subscapular abscesses due to multiple reasons. These infections are rare, deep-seated, with mild clinical features, leading to delayed diagnosis [2,4,6-8,12]. Misdiagnosis as septic arthritis and frozen shoulder are common. Differentials include tumors, traumatic conditions, and degenerative arthritis [6,12,13,17,24]. Subscapular abscesses should be considered in cases of shoulder pain with or without fever or elevated blood markers [6]. Delayed diagnosis can result in the spread of infection, pneumonia, meningitis, and septicemia [3,4,9]. Abscess identification by imaging is key to early diagnosis. MRI and CT are the best modalities, as false-negative reports are not unusual with ultrasonograms [15,19].

Our case represents the oldest reported at 83 years of age, with the next oldest patient being 72 years. Streptococcus has been reported as the causative organism only once before [4]. This is the first case caused by GBS (Streptococcus agalactiae) and the first reported invasive GBS (IGBS) subscapular abscess.

GBS infections most commonly affect neonates [25,26] but have recently been on the rise among the elderly [26]. Elderly GBS infections usually occur in immunocompromised patients with multiple comorbidities and are twice as common in males [25,26]. IGBS usually presents as bacteremia (50%) and pneumonia (25%) [26]. However, in this case, the patient had a urinary tract infection and subscapular abscess. More than 50% of IGBS cases occur in individuals older than 70 years. Ninety percent of IGBS-related deaths [25-27] occur within this age group, highlighting the importance of early identification and treatment. When dealing with an aggressive staphylococcal subscapular infection in a patient with a history of recurrent skin infections, Panton-Valentine leukocidin (PVL)-producing staphylococci should be suspected. To minimize the associated higher complication rates, specific antibiotics with PVL antitoxin effect, such as clindamycin, linezolid, or rifampicin, should be used [17,20].

The history of trauma during physiotherapy, increased bleeding tendency due to anticoagulant therapy, and evidence of hemosiderin on MRI suggest that the abscess evolved from a hematoma. The presence of *Streptococcus agalactiae* in the urine and respiratory cultures points to these as the seeding source. Trauma from physiotherapy leading to a hematoma [16,24,28] and hematoma seeding leading to abscess [16,17] have been described before.

Key principles of treatment are early diagnosis with a high index of suspicion and proper imaging followed by drainage with concurrent appropriate antibiotics [6,7]. Drainage may be percutaneous image-guided [3,18,22] or surgical. It is optimal to choose the surgical approach for drainage based on abscess location and extent. The axillary approach is best suited for an abscess in the subscapular space pointing to the axilla without glenohumeral or subscapularis muscle involvement. In predominant involvement of the subscapularis muscle with or without glenohumeral infection, an anterior deltopectoral approach would be appropriate as it will allow both issues to be addressed. Grange et al. [15] combined a deltopectoral approach with shoulder arthroscopy to address the glenohumeral joint. Subcapsular space access is more difficult with an anterior deltopectoral approach. If the subscapular space infection tracks to the axilla and then inferiorly to the arm or flank, a dual approach may be considered, as described by Pushpasekaran et al. [21]. They performed a deltopectoral approach coupled with a second incision over the anterior aspect of the posterior axillary fold, inferolateral to the scapula, and dissected anterior to the latissimus dorsi.

Abscesses situated predominantly in the subscapular or scapulothoracic space without subscapular muscle or glenohumeral involvement are amenable to posterior approaches. Posterior approaches have evolved with time and may be posteromedial, posterolateral, or combined. Earlier approaches were posteromedial, including the Judet approach [14], posteromedial with release of the rhomboids [1], posteromedial with cutting of the trapezius [8], and along the medial two-thirds of the scapula [7], all of which resulted in muscle damage. In the posterolateral approach by Khaw and Faisham [24], the teres major, teres minor, and infraspinatus muscles were detached along the lateral border of the scapula. Christman-Skieller et al. [10] used a muscle-sparing posterolateral approach in the inter-nervous plane between latissimus dorsi and teres major, combined with medial counter incisions and stab wounds through rhomboid major and serratus anterior muscles. For our case, as the bulk of the abscess was in the subscapular space pointing laterally with no glenohumeral joint or subscapularis muscle involvement, we opted for a modification of the combined approach of Christman-Skieller et al. [10]. We believe that the counter incisions are not necessary if there is adequate access to the medial side through the posterolateral approach. In obese or muscular patients, however, counter incisions may be required.

## Conclusions

Subscapular abscesses are a rare but serious condition requiring a high index of suspicion for early diagnosis. MRI with contrast is a dependable imaging modality that sheds light on the diagnosis and helps in planning surgical intervention. The choice of approach for drainage depends on the location and extent of the abscess. In the elderly population, timely diagnosis and treatment prevent complications and mortality. This is the first report of IGBS - *Streptococcus agalactiae* infection in a subscapular abscess. We have also highlighted the usage of a muscle-sparing approach for optimal outcome and compared our management with previously described cases.

## References

[1] Nowinski RJ, Duchene C (2004). Spontaneous septic subscapular abscess. A case report. J Bone Joint Surg Am.

[2] East J, Piper D, Chan S (2020). Spontaneous intramuscular abscesses involving the rotator cuff muscles in two cases presenting during the COVID-19 pandemic. Cureus.

[3] Indra FIPBD, Akbar SIBWM, Ibrahim MARB, Han OL (2022). A rare case of isolated post-traumatic subscapularis abscess in a paediatric patient. Bali Med J.

[4] Furuhata R, Inoue D, Kiyota Y, Morioka H, Arino H (2019). Dorsal subscapularis approach for the surgical drainage of subscapularis intramuscular abscess: a case report. BMC Musculoskelet Disord.

[5] Handorf CR (1983). Fatal subscapular staphylococcal abscess. South Med J.

[6] McFarlane IV, Wong M, Alder-Price AC (2024). Subscapular abscesses: a literature review and evidence-based treatment guidelines. Shoulder Elbow.

[7] Mourkus H, Vadivelu R, Phillips J (2018). Literature review and a case report of spontaneous subscapular abscess in a child. Eur J Orthop Surg Traumatol.

[8] Saxena P, Konstantinov IE, Zelei D, Newman MA (2008). Spontaneous subscapular abscess: a rare surgical condition. Heart Lung Circ.

[9] Yilmaz G, Standard SC (2012). Periscapular abscess: unusual cause of shoulder pain in children. J Pediatr Orthop B.

[10] Christman-Skieller C, McIntyre LK, Plevin R, Friedrich JB, Smith DG (2017). A posterolateral approach to the scapula for evacuation of a subscapular abscess: a case report. JBJS Case Connect.

[11] (2024). Connected Papers | Find and explore academic papers. https://www.connectedpapers.com/about.

[12] Babayiğit A, Makay B, Demircioğlu F, Cakmakçi H, Unsal E (2009). Subscapular abscess after blunt trauma. Pediatr Emerg Care.

[13] Fernández Pérez M, Orradre Burusco I, Mondragón Rubio J, Azcona Martínez de Baroja L, Romero Redondo I, Cornejo Jiménez D (2020). Spontaneus subscapular abscess. A clinical case. An Sist Sanit Navar.

[14] Giugale JM, Bosch PP, Grudziak JS (2015). Subscapular abscess in a nine-year-old female patient: a case report. JBJS Case Connect.

[15] Grange S, Mohamed Yousif Mohamed A, Salih M, Meda MR (2024). Subscapularis pyomyositis, a rare cause of shoulder pain, in a patient without apparent risk factors: a case report. Int J Surg Case Rep.

[16] Ham DH, Wang S (2022). Escherichia coli subscapular abscess as a rare complication of manual therapy in frozen shoulder: a case report. Am J Surg Case Rep.

[17] Jagernauth S, Clough RA, Noorani A, Ahmad M (2018). Subscapularis pyomyositis: a rare presentation of shoulder pain. BMJ Case Rep.

[18] Koratala A, Alquadan KF, Chornyy V, Qadri I, Ejaz AA (2017). Subscapular abscess associated with buttonhole cannulation technique of arteriovenous fistula for hemodialysis access. J Vasc Access.

[19] Park IK, Jang SH (2020). Subscapularis pyomyositis presenting as shoulder stiffness mistaken as frozen shoulder in young female: a case report. Radiol Case Rep.

[20] Patel K, Spowart E, Sochorova D, Diego N, Mamarelis G, Sohail MZ (2018). Subscapular abscess caused by Panton-Valentine leukocidin-positive Staphylococcus aureus: an atypical presentation. Case Rep Orthop.

[21] Pushpasekaran N, Selvakkalanjiyam S, Rajesh MK, Sivanandan MH, Sundaram KM (2024). Shoulder girdle muscle abscess: Potential routes of spread and surgical management by a dual anterior approach. J ISAKOS.

[22] Ren H, Memauri B, Sharma A (2021). Disseminated blastomycosis causing scapular destruction. CMAJ.

[23] San Joaquin VH, Kimball JB (1980). Subscapular abscess due to Haemophilus influenzae type B. Pediatrics.

[24] Khaw YC, Faisham WI (2019). A single posterolateral scapular approach to drain post-traumatic intramuscular scapular and axillary abscess in an adolescent: a case report. Malays Orthop J.

[25] Lopardo HA, Vidal P, Jeric P, Centron D, Paganini H, Facklam RR, Elliott J (2003). Six-month multicenter study on invasive infections due to group B streptococci in Argentina. J Clin Microbiol.

[26] Morozumi M, Wajima T, Takata M, Iwata S, Ubukata K (2016). Molecular characteristics of group B streptococci isolated from adults with invasive infections in Japan. J Clin Microbiol.

[27] Parks T, Barrett L, Jones N (2015). Invasive streptococcal disease: a review for clinicians. Br Med Bull.

[28] Tzaveas A, Anastasopoulos N, Paraskevas G, Natsis K (2016). A rare case of quadratus femoris muscle rupture after yoga exercises. Clin J Sport Med.

